# Glutathione Peroxidase 8 Suppression by Histone Deacetylase Inhibitors Enhances Endoplasmic Reticulum Stress and Cell Death by Oxidative Stress in Hepatocellular Carcinoma Cells

**DOI:** 10.3390/antiox10101503

**Published:** 2021-09-22

**Authors:** Hae-Ahm Lee, Ki-Back Chu, Eun-Kyung Moon, Fu-Shi Quan

**Affiliations:** 1 Medical Research Center for Bioreaction to Reactive Oxygen Species and Biomedical Science Institute, School of Medicine, Graduate School, Kyung Hee University, Seoul 02447, Korea; halee23@khu.ac.kr; 2 Department of Biomedical Science, Graduate School, Kyung Hee University, Seoul 02447, Korea; kbchu@khu.ac.kr; 3 Department of Medical Zoology, School of Medicine, Kyung Hee University, Seoul 02447, Korea; ekmoon@khu.ac.kr

**Keywords:** GPX8, HCC, ER stress, HDACi, oxidative stress, apoptosis

## Abstract

Histone deacetylase inhibitors (HDACi) are emerging as anti-hepatocellular carcinoma (HCC) agents. However, the molecular mechanisms underlying HDACi-induced sensitization to oxidative stress and cell death of HCC remain elusive. We hypothesized that HDACi reduces the anti-oxidative stress capacity of HCC, rendering it more susceptible to oxidative stress and cell death. Change in the transcriptome of HCC was analyzed by RNA-seq and validated using real-time quantitative polymerase chain reaction (qPCR) and Western blot. Cell death of HCC was analyzed by fluorescence-activated cell sorting (FACS). Protein localization and binding on the target gene promoters were investigated by immunofluorescence (IF) and chromatin immunoprecipitation (ChIP), respectively. Glutathione peroxidase 8 (GPX8) was highly down-regulated in HCC upon oxidative stress and HDACi co-treatment. Oxidative stress and HDACi enhanced the expression and transcriptional activities of ER-stress-related genes. N-acetyl-cysteine (NAC) supplementation reversed the oxidative stress and HDACi-induced apoptosis in HCC. HDACi significantly enhanced the effect of ER stressors on HCC cell death. GPX8 overexpression reversed the activation of ER stress signaling and apoptosis induced by oxidative stress and HDACi. In conclusion, HDACi suppresses the expression of GPX8, which sensitizes HCC to ER stress and apoptosis by oxidative stress.

## 1. Introduction

The global hepatocellular carcinoma (HCC) incidence has been on the rise over the past decade, and the mortality associated with it has been reported to be the second highest among all cancer-related deaths [1]. HCC is the most dominant form of primary liver cancer, accounting for approximately 90% of all clinical primary liver cancer cases [2]. Since clinical manifestations in the early stages of HCC tend to be asymptomatic and are difficult to discern, most HCC patients are diagnosed at the advanced stage, which limits available treatment options [3]. While therapeutic interventions are possible, chemoresistance development in HCC has rendered the drugs ineffective [4]. In 2007, based on the positive results acquired from the Sorafenib Hepatocellular Carcinoma Assessment Randomized Protocol (SHARP) trial, sorafenib was approved by the US Food and Drug Administration (FDA) as a first-line treatment option for advanced HCC [5]. At present, several other drugs, such as lenvatinib, regorafenib, and cabozantinib, have been developed and approved by the FDA for treating unresectable HCC [6]. While sorafenib treatment can prolong the survival of advanced HCC patients, its efficacy is hampered by drug resistance, which can be attributed to the high degree of heterogeneity in HCC [7]. With the rising chemoresistance in HCC becoming a major hindrance for treatment, identifying alternative drugs and biomarkers and determining an efficacious therapeutic regimen are imperative [4,7].

Histone deacetylase (HDAC) is an epigenetic modulator that removes acetyl groups from the histone tail to result in transcriptionally inactive heterochromatin. Small molecules for HDAC inhibition that reverse aberrant epigenetic changes related to cancer have shown potent anticancer effects in a large number of studies [8]. Several HDAC inhibitors (HDACi), such as romidepsin, vorinostat, velinostat, and Panobinostat, have been approved by the US FDA in the past decades for T cell lymphoma treatment [9]. Gene expression of several HDAC isoforms was reported to be significantly elevated in HCC cell lines and tissues in comparison to their respective counterparts, which highlights the potential of HDACs as therapeutic targets in HCC [10]. To this end, preclinical and phase I/II clinical trials involving HDACi alone or in combination with sorafenib for HCC treatment have been conducted [11,12,13]. While unresolved HDACi-associated issues continue to persist, including but not limited to a broad spectrum of HDAC isoform inhibition, non-selective or genome-wide modulation of gene expressions, and side effects, the use of HDACi alone or combined with other anticancer drugs remains an attractive therapeutic option for HCC.

Reactive oxygen species (ROS) are a by-product of cellular metabolism. Physiological concentrations of ROS play a role in numerous cell functions, including proliferation, differentiation, migration, and angiogenesis. A higher concentration of ROS sequentially results in stress response, adaptation, inflammatory response, growth arrest, and finally cell death in normal cells as well as tumor cells [14]. Tumor cells produce greater quantities of ROS than normal cells as a consequence of an augmented metabolic rate and genome instability, which are detoxified by increased antioxidant enzymes and molecules in cells [15]. Ironically, the heightened redox balance in tumor cells makes them more vulnerable to oxidative stress accumulation induced through either increased ROS generation or decreased ROS-scavenging ability [16]. Thus, many ROS-producing agents, such as procarbazine, doxorubicin, and rapamycin, have been used in clinical settings for treating various cancers [15].

In mammalian cells, antioxidant systems have been developed to protect cells from oxidative stress. The most important ROS-scavenging enzyme systems are catalase (CAT), peroxiredoxin (PRDX), and glutathione peroxidases (GPXs). CAT directly reduces H_2_O_2_ to H_2_O and O_2_ in the peroxisome. PRDX uses thioredoxin to reduce H_2_O_2_ [17]. The GPX family uses glutathione (GSH) to reduce substrates. To date, eight GPX isoforms have been classified based on their structural similarities [18]. GPX isoforms containing selenocysteine in their active site (GPX1–4 and GPX6) can couple with GSH, whereas cysteine-containing isoforms (GPX5, GPX7, and GPX8) do not have GSH-binding affinity [19]. The localization of each GPX isoform also varies. GPX1 is localized in the cytosol and mitochondria, while GPX2 primarily resides in the cytosol and nucleus. GPX3 and GPX5 are located in the extracellular matrix, and GPX4 is predominantly found in the nucleus and plasma membrane. GPX7 and GPX8 are the only isoforms observed in the endoplasmic reticulum (ER).

Recently, we demonstrated that HDACi treatment sensitizes HCC cells to oxidative stress and cell cycle arrest [20]. However, the molecular mechanisms underlying HDACi-induced sensitization of HCC cells to oxidative stress are still unclear. In the present study, we hypothesized that HDACi reduces the anti-oxidative stress capacity of HCC, which results in oxidative-stress-sensitive HCC.

## 2. Materials and Methods

### 2.1. Antibodies, Plasmids, and Chemicals

Antibodies to detect human pancreatic ER kinase (PERK), eukaryotic translation initiation factor 2a (eIF2a), activating transcription factor (ATF)-3, ATF-4, C/EBP homologous protein (CHOP), cation transport regulator homolog 1 (CHAC1), and caspase 12 were purchased from Cusabio (Hubei, China). Anti-phospho-PERK, p-eIF2a, glutathione peroxidase 8 (GPX8), and glyceraldehyde 3-phosphate dehydrogenase (GAPDH) were purchased from Santa Cruz Biotechnology, Inc. (Dallas, TX, USA). MS-275, suberanilohydroxamic acid (SAHA), tertiary-butyl-hydroperoxide (tBHP), tunicamycin, and thapsigargin were purchased from Tocris Bioscience (Bristol, UK). Cell-Counting Kit 8 (CCK8) was purchased from Dojindo Molecular Technologies (Rockvill, MD, USA). The apoptosis detection kit was purchased from BD Biosciences (Franklin Lakes, NJ, USA). GPX8 rescue PLAK1 was a gift from Prof. Yoav Shaul (Addgene plasmid #161517).

### 2.2. Cell Culture and Cell Viability Test and Animal Ethics

Six-week-old Sprague–Dawley rat primary hepatocytes were isolated using collagenase (types II and IV). Primary hepatocytes, HepG2 and Hep3B cells, were cultured in Dulbecco’s Modified Eagle’s Medium (DMEM) supplemented with 10% fetal bovine serum (FBS) and 1% penicillin/streptomycin at 37 °C with 5% CO_2_. To check cell viability, cells (5 × 10^3^) were seeded in a 96-well plate with 100 μL of medium. The next day, the cells were treated with agents such as tBHP (~100 μmol/L), SAHA (1 μmol/L), MS-275 (1 μmol/L), tunicamycin (~1 μmol/L), and thapsigargin (~1 μmol/L) either alone or combined with other reagents. After 22 h, 10 μL of CCK8 was added to each well and incubated for 2 h. Optical density was measured with EZ Read 400, a microplate reader (Biochrom Ltd., Cambridge, UK) at 450 nm. All experimental procedures involving animals were approved and conducted following the guidelines set out by the Kyung Hee University IACUC (permit no. KHSASP-20-213).

### 2.3. RNA Extraction and RNA-Seq

Cells (1 × 10^6^) were seeded in 100 mm culture dishes. The next day, the cells were treated with tBHP (25 μmol/L), SAHA (1 μmol/L), or tBHP and SAHA co-treatment. After 24 h, total RNA was extracted by using the RNAeasy mini kit (Qiagen, Venlo, the Netherlands). Massive Analysis of cDNA End (MACE) was conducted, as described previously [20]. Briefly, a cDNA library was constructed using the QuantSeq 3′ mRNA-Seq Library Prep Kit (Lexogen, Inc., Vienna, Austria). High-throughput sequencing was performed by using NextSeq 500 (Illumina, Inc., San Diego, CA, USA). Bowtie2 was used to align the QuantSeq 3′ mRNA-Seq Library Prep Kit. The normalized read count (NRC) was calculated using Edge R within R (R development core team, 2016). A heatmap of differentially expressed genes was generated with Multiple Experiment Viewer (MeV) software. Fastq from MACE was deposited in the National Center for Biotechnology Information (NCBI) Sequence Read Archive (SRA) repository (accession no. PRJNA678827; https://www.ncbi.nlm.nih.gov/Traces/study/?acc=PRJNA678827) (accessed on 6 April 2021).

### 2.4. Quantitative Real-Time Polymerase Chain Reaction (qPCR)

The qPCR experiment was performed according to the Minimum Information for Publication of Quantitative Real-Time PCR Experiments (MIQE) guidelines [21]. After total RNA extraction by using the RNeasy mini kit (Qiagen), cDNA synthesis was performed by using the RevertAid^TM^ First Strand cDNA synthesis kit (Fermentas, Vilnius, Lithuania) following the manufacturer’s instructions. Briefly, 2 μg of RNA and 1 μL of a random hexamer were incubated at 65 °C for 5 min and placed on ice. Then, 1X reaction buffer, 20 U RNAse inhibitor, 1 mmol/L of dNTP, and 200 U reverse transcriptase were added. The cDNA synthesis reactions were as follows: 5 min at 25 °C, 60 min at 42 °C, and 5 min at 72 °C. The synthesized cDNA was diluted 10-fold to use as a template for qPCR. The reaction volume for qPCR was set to 20 μL, which included the Luna^®^ Universal qPCR Master Mix (10 μL, NEB, Ipswich, MA, USA), cDNA (2 μL), a primer set (100 nmol/L each), and pure water up to 20 μL. Then qPCR was conducted using micPCR (PhileKorea, Seoul, Korea). PCR conditions were as follows: 2 min at 95 °C and 40 cycles at 95 °C for 15 s followed by 1 min at 60 °C. Primer specificity was confirmed by dissociation curves after PCR and insufficiently amplified values were removed by qPCR software. The cycle of critical threshold (Ct) value was normalized to that of *Gapdh* because its expression level was the most abundant (the lowest Ct) in HCC and insignificantly changed by treatments in the present study. All primer sets used in the present study are shown in Table S1.

### 2.5. Fluorescence-Activated Cell Sorting (FACS)

HCC cells (5 × 10^5^) were seeded in 60 mm culture dishes. The next day, the cells were treated with tBHP (25 μmol/L), tunicamycin (0.25 μmol/L), thapsigargin (0.25 μmol/L), NAC (100 μmol/L), and HDACi (1 μmol/L) alone or combined. After 24 h, the cells were harvested with trypsin/EDTA and then washed with cold PBS two times. Cells were stained using the FITC–Annexin V Apoptosis Detection Kit (BD Pharmigen^TM^, Franklin Lakes, NJ, USA). Afterward, stained cells were acquired by a C6 Accuri flow cytometer and analyzed using FlowJo software (BD Biosciences, Franklin Lakes, NJ, USA).

### 2.6. Western Blotting

HCC cells (5 × 10^5^) were seeded in 60 mm dishes, and the next day, the cells were treated with reagents for 24 h. Cell lysates were extracted using Pro-Prep lysis buffer (Intron, Daejeon, Korea) containing PMSF (1 mmol/L), EDTA (1 mmol/L), pepstatin A (1 mmol/L), leupeptin (1 mmol/L), and aprotinin (1 μM) for protease inhibition. An Xpert phosphatase inhibitor cocktail (GenDEPOT, Katy, TX, USA) was added to this buffer for phosphatase inhibition. Protein concentrations were determined by Bradford assay (Bio-Rad Laboratories, Hercules, CA, USA). Cell lysates (30 μg) were separated on SDS-PAGE and transferred to a nitrocellulose (NC) membrane. After blocking the membrane with 5% skim milk for 1 h, the membrane was incubated with a primary antibody (0.1~0.5 μg/mL) at 4 °C, overnight. The membrane was washed with TBST buffer (25 mmol/L Tris base, 150 mmol/L NaCl, and 0.1% Tween 20) three times and then incubated with a secondary antibody (1:5000) for 1 h. After washing with TBST buffer three times for 10 min, the membrane was incubated with an enhanced chemiluminescence (ECL) agent (Thermo Fisher Scientific, Waltham, MA, USA). Band images were captured by the ChemiDoc Imaging system (Bio-Rad Laboratories, Hercules, CA, USA). For semi-quantitative analyses of the Western blot results, band densities were determined by using the ImageJ program (NIH) and Image Lab software (Bio-Rad Laboratories). The density values of target genes were normalized to that of the endogenous control (GAPDH). The mean and SD values were calculated from normalized density values.

### 2.7. Immunofluorescence (IF)

Protein localization was investigated by IF. HCC cells were cultured on cover glasses in 6-well plates. The next day, the cells were treated with tBHP (25 μmol/L), SAHA (1 μmol/L), or both. After 24 h, the cells were fixed with ethanol at −20 °C for 1 h. The fixed cells were hydrated by washing with PBS twice. The cells were blocked with 1% bovine serum albumin in TBST for 1 h. A pimary antibody (0.5 μg/mL) was added and incubated overnight at 4 °C. After washing with PBST three times for 10 min each, the cells were incubated with a fluorescein (FITC)-conjugated secondary antibody (1:200) for 1 h. The cells were washed with PBST three times for 10 min each, and cell nuclei were stained with 4′,6-diamidino-2-phenylindole (DAPI). Cell images were captured by fluorescent microscopy (Leica Microsystems, Wetzlar, Germany).

### 2.8. Chromatin Immunoprecipitation (ChIP)

Enrichment of the transcription factor (TF) on the target gene promoters was investigated by ChIP assay using the EpiTect ChIP OneDay Kit (Qiagen, Venlo, The Netherlands) following the manufacturer’s instructions with minor modifications. Briefly, formalin-fixed cells were lysed with SDS lysis buffer (1% SDS, 10 mM EDTA, and 50mM Tris, pH 8.1) including an Xpert protease inhibitor cocktail (GenDEPOT, Katy, TX, USA). To shear chromatin to an average length of about 200–500 bp, cell lysates were sonicated using a sonicator (Branson-Emerson, MO, USA). Sheared chromatin was pre-cleared with protein agarose beads at 4 °C for 1 h and then incubated with a primary antibody (2 μg) overnight at 4 °C. The protein–antibody complex was pulled down with the protein agarose bead at 4 °C for 1 h and sequentially washed with a low-salt solution, a high-salt solution, LiCl solution, and Tris-EDTA solution twice. DNA was eluted after incubating with elution buffer (1% SDS and 0.1 mol/L NaHCO_3_) containing protease K at 45 °C for 30 min using DNA-binding beads and columns. TF binding on the target gene promoters was analyzed by qPCR. Primer sets for ChIP assay are shown in Table S2.

### 2.9. Glutathione Assay

The intracellular glutathione level was analyzed by using the EZ-Glutathione Assay Kit (DoGenBio, Seoul, Korea) following the manufacturer’s instructions. HCC cells (1 × 10^5^) were seeded in a 6-well plate, and the next day, the cells were treated with tBHP, SAHA, or both. After 24 h, the cells were washed twice with PBS and then lysed with Pro-Prep solution (Intron Biotechnology, Daejun, Korea). To assess the oxidized form of glutathione (GSSG), free thiol groups were scavenged with 1-methyl-2-vinylpyridinium trifluoromethanesulfonate1 (M2VP). Masked cell lysates were diluted with 5% metaphosphoric acid (MPA). For reduced glutathione (GSH) assay, cell lysates were diluted with 5% MPA solution without masking. Samples were diluted with MPA and incubated with 5,5-dithio-bis-2-nitrobenzoic acid (DTNB) and glutathione reductase (GR) in a 96-well plate at room temperature for 5 min. Nicotinamide adenine dinucleotide phosphate (NADPH) solution was added to each well, and enzyme kinetics were analyzed by measuring OD_412nm_ values for 3 min.

### 2.10. Statistics

Graphs are expressed as mean±SD. One-way analysis of variance (ANOVA) followed by Dunnett’s post hoc comparison was applied for data analysis. Statistical significance was considered at *p* < 0.05. Student’s *t*-test was applied for analyzing significant differences between the two groups.

## 3. Results

### 3.1. HDACi Repressed Expression of GPX8 in the HCC

Previously, we demonstrated that HDACi treatment sensitizes HCC cells to oxidative stress and cell cycle arrest [20]. Thus, we speculated that the antioxidant system in HCC was downregulated by HDACi treatment. MACE result revealed more than two-fold reduction in several antioxidant enzymes, notably glutathione peroxidase 8 (GPX8), microsomal glutathione S-transferase 1 and 2 (MGST1 and MGST2), glutathione S-transferase omega 1 (GSTO1), NAD(P)H quinone oxidoreductase 1 (NQO1), peroxiredoxin 3 (PRDX3), thioredoxin reductase 2 (TXNRD2), peroxidasin (PXDN), and thioredoxin (TXN) upon SAHA or combinatorial treatment involving both tBHP and SAHA in HepG2 cells.

**Figure 1:**
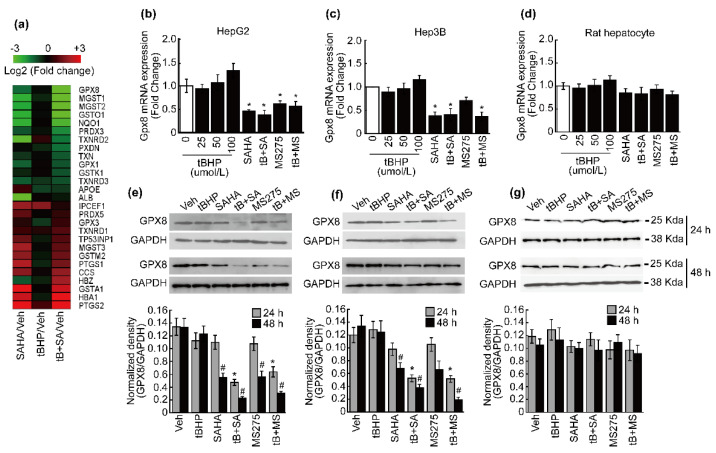
Suppression of GPX8 by oxidative stress and HDACi in HCC. (**a**) Heatmap of antioxidant enzymes with expression changes exceeding 2-fold upon SAHA or combined treatment with tBHP and SAHA in HepG2 cells. (**b**) Expression of *Gpx8* mRNA was insignificantly changed by tBHP, but significant reductions were induced by HDACi and HDACi combined with tBHP. (**c**) Expression of *Gpx8* mRNA was negligibly changed by tBHP but significantly decreased by SAHA and combinatorial treatment with tBHP and HDACi in Hep3B cells. (**d**) *Gpx8* mRNA was negligibly changed by tBHP, HDACi, and co-treatment in rat hepatocytes. Protein level of GPX8 was analyzed by Western blotting. At 24 h after treatment, altered GPX8 expression was only observed in cells receiving combined treatment with HDACi and tBHP, while significant downregulation from a single treatment with HDACi became evident 48 h after treatment in HepG2 (**e**) and Hep3B (**f**) cells. (**g**) GPX8 protein level was negligibly changed by tBHP, HDACi, or combined treatment in rat hepatocytes. All graphs were presented as the mean ± SD of three independent experiments (*^, #^ *p* < 0.05 vs. vehicle).

In particular, compared to vehicle-treated HepG2 cells, Gpx8 expression was decreased 4- and 10-fold upon SAHA and combinatorial treatment, respectively. Furthermore, unlike other antioxidant enzymes, combining tBHP with SAHA synergistically reduced GPX8 expression (Figure 1a). The MACE result for Gpx8 expression was validated by qPCR. Gpx8 expression was negligibly changed by tBHP treatment, and HDACi (SAHA and MS275) treatment significantly decreased the expression of Gpx8 mRNA in HepG2 cells regardless of tBHP presence (Figure 1b). Similar GPX8 expression patterns were observed in another HCC cell line. While tBHP-induced changes to Gpx8 mRNA expression in Hep3B cells were negligible, HDACi substantially reduced the expression of Gpx8 irrespective of tBHP (Figure 1c). GPX8 mRNA expression was negligibly changed by tBHP, HDACi, or combinatorial treatment in normal hepatocytes (Figure 1d). Noticeable reduction in the GPX8 protein level was observed in HepG2 cells receiving combinatorial treatment after 24 h, and this phenomenon was also observed in single HDACi-treated cells after 48 h (Figure 1e). Consistent with the results of HepG2 cells, combinatorial treatment with tBHP and HDACi effectively reduced the GPX8 protein level in Hep3B cells, whereas neither tBHP nor HDACi alone had any noticeable effect at 24 h. GPX8 protein reduction became more evident as time progressed, with HDACi treatment alone inducing significant reduction at 48 h post-treatment (Figure 1f). The GPX8 protein level was hardly changed by treatment with tBHP, HDACi, or combinatorial treatment in rat hepatocytes (Figure 1g).

### 3.2. HDACi Induced Endoplasmic Reticulum Stress (ER Stress)

**Figure 2:**
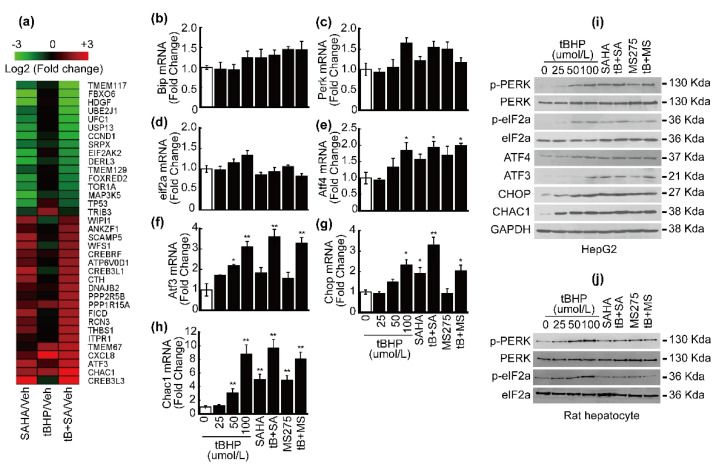
Co-treatment with tBHP (25 μmol/L) and HDACi (1 μmol/L) elevated ER stress gene expression. (**a**) Heatmap of ER-stress-related genes whose expression changed more than 2-fold by SAHA or combined treatment with tBHP and SAHA in HepG2 cells. Expression of *Bip* (**b**), *Perk* (**c**), and *eif2a* (**d**) negligibly changed upon tBHP, HDACi, and combined treatment with both. (**e**) *Atf4* mRNA expression significantly increased at a high concentration of tBHP (100 μmol/L) and combined treatment with tBHP and HDACi. (**f**) *Atf3* mRNA expression significantly increased by tBHP in a dose-dependent manner. Co-treatment with tBHP and HDACi significantly elevated the expression of *Atf3* mRNA. (**g**) *Chop* mRNA expression significantly increased by a high concentration of tBHP (100 μmol/L), SAHA, and combined treatment with tBHP and HDACi. (**h**) Expression of *Chac1* increased by tBHP in a dose-dependent manner. *Chac1* mRNA expression was induced upon a single treatment with HDACi as well as combined treatment. (**i**) Incremental, though not significant, changes to total PERK and eIF2a expression were induced by tBHP and/or HDACi, whereas phosphorylated forms of PERK and eIF2a dose-dependently increased by tBHP. A single treatment with HDACi increased the expression of phosphorylated PERK and eIF2a, which synergistically increased by combined treatment with tBHP and HDACi. Protein levels of ATF4, ATF3, CHOP, and CHAC1 increased by tBHP in a dose-dependent manner. The expression of these proteins was elevated by HDACi treatment alone, which synergistically increased by combinatorial treatment in HepG2 cells. (**j**) Phosphorylation levels of PERK and eIF2a dose-dependently increased by tBHP but were unaffected by HDACi or combinatorial treatment in rat hepatocytes. Data were presented as the mean ± SD of three independent experiments. (* *p* < 0.05, ** *p* < 0.01 vs. vehicle).

Since GPX8 is an ER-specific antioxidant enzyme whose expression decreased through HDACi treatment in HCC cell lines, we analyzed the expression of ER-stress-related genes in MACE results to confirm the correlation between ER stress and HDACi. Atf3 expression negligibly changed by a single treatment with tBHP, but its expression was enhanced 4-fold and 6-fold following SAHA and combinatorial treatment, respectively. Chac1 expression also underwent similar changes, with SAHA and combinatorial treatment inducing 4-fold and 10-fold increases, respectively (Figure 2a). Although Chop was not displayed on the heatmap, the MACE result showed that the expression of Chop increased approximately 2.5-fold by combinatorial treatment with tBHP and SAHA (data not shown). Next, we focused on the PERK/eIF2a/ATF4/CHOP pathway because CHOP and CHAC1 are some of the final effectors of this pathway [22]. The expression of *Bip* (Figure 2b), *Perk* (Figure 2c), and *eif2a* (Figure 2d) mRNA levels negligibly changed by tBHP, HDACi, or combinatorial treatment with tBHP and HDACi. The expression of Atf4 (Figure 2e) and Atf3 (Figure 2f) significantly increased by a high concentration of tBHP (100 μmol/L) and combinatorial treatment involving both tBHP and HDACi. A high concentration of tBHP (100 μmol/L), a single treatment with SAHA, and combinatorial treatment with both elevated the expression of Chop. Interestingly, the effects of a single treatment with MS-275 in HepG2 cells were negligible (Figure 2g). The expression of Chac1 significantly increased post-treatment with 50 μmol/L of tBHP, HDACi alone, or both (Figure 2h). The protein expression of ER-stress-related genes was investigated by Western blot. While tBHP, HDACi, or co-treatment failed to elicit any changes in total protein levels of PERK and eIF2a, drastic increases in phosphorylated PERK and eIF2a expression were induced by treatment with a high concentration of tBHP (50 μmol/L~), HDACi alone, and the two combined. Similarly, ATF4 and ATF3 protein expression was also enhanced through a high concentration of tBHP, a single treatment with HDACi, and combinatorial treatment with tBHP and HDACi. Enhanced CHOP and CHAC1 induction was observed even at low concentrations of tBHP (25 μmol/L~) and also in both HDACi and co-treated HepG2 cells (Figure 2i). Phosphorylation levels of PERK and eIF2a were dose-dependently elevated by tBHP, which was insignificantly affected by HDACi or co-treatment with tBHP and HDACi in rat hepatocytes (Figure 2j).

### 3.3. Co-Treatment with tBHP and HDACi Enhanced Transcriptional Activity of ATF4 and CHOP

**Figure 3:**
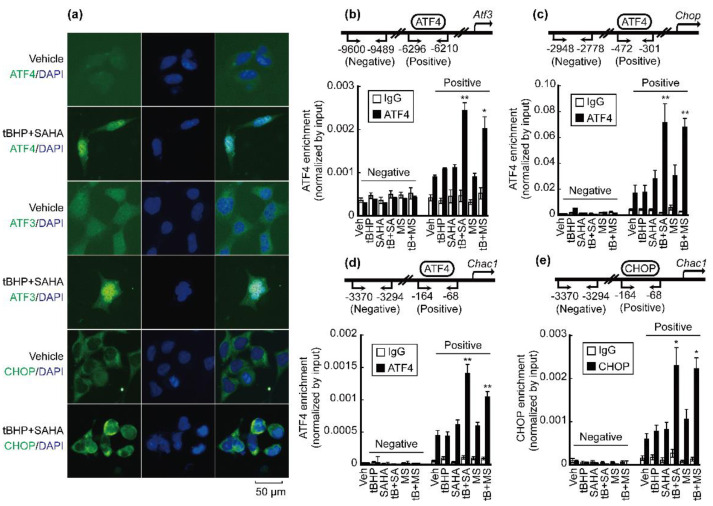
Transcriptional activity of ATF4 and CHOP increased by combined treatment with tBHP and HDACi. (**a**) Nuclear translocation of ATF4, ATF3, and CHOP was investigated by IF. ATF4, ATF3, and CHOP were mainly localized in the cytosol, which were translocated into the nuclei after treatment with tBHP and SAHA. Promoter-binding affinity was tested by ChIP assay. (**b**) ATF4-binding affinity on the promoter region of *Atf3* significantly increased by co-treatment with tBHP and HDACi such as SAHA or MS275. (**c**) Enrichment of ATF4 on the promoter region of *Chop* significantly increased by co-treatment with tBHP and HDACi. (**d**) ATF4 binding on the promoter of *Chac1* was significantly elevated by combined treatment with tBHP and HDACi. (**e**) CHOP, which occupies the identical binding site on the promoter region of *Chac1* as ATF4, was enriched following tBHP and HDACi combinatorial treatment. Data were presented as the mean ± SD of three independent experiments. (* *p* < 0.05, ** *p* < 0.01 vs. vehicle).

Immunoflulorescence (IF) was performed to confirm the nuclear translocation of ATF4, ATF3, and CHOP. ATF4, ATF3, and CHOP proteins were mainly dispersed in the cytosol of HepG2 cells. Combined treatment with tBHP and SAHA induced translocation of these proteins from the cytosol into the nuclei of HepG2 cells (Figure 3a). Binding of ATF4 and CHOP on the promoters of downstream genes was analyzed by chromatin immunoprecipitation (ChIP) assay. ATF4 is a well-known transcription factor of ATF3, CHOP, and CHAC1 [22]. A single treatment with tBHP, SAHA, or MS275 marginally enriched ATF4 binding on the promoters of target genes such as Atf3, Chop, and Chac1. Combined treatment with tBHP and HDACi increased the enrichment of ATF4 on the promoter of Atf3 (Figure 3b), Chop (Figure 3c), and Chac1 (Figure 3d). Similar findings were observed from the enrichment of CHOP on the promoter of Chac1. A single treatment with tBHP or HDACi showed a negligible effect on CHOP binding on the promoter of Chac1, which significantly increased by combining the two reagents (Figure 3e).

### 3.4. HDACi Enhanced Oxidative Stress and ER-Stress-Induced Apoptosis of HCC Cells

**Figure 4:**
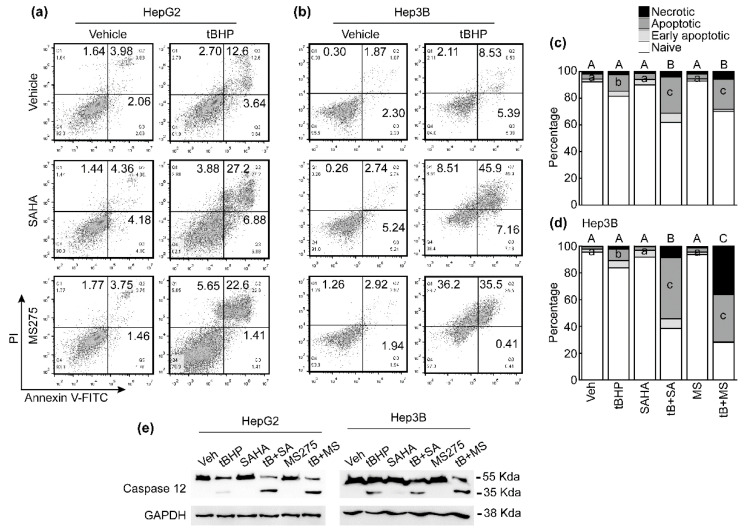
Combined treatment with tBHP and HDACi induced apoptosis in HCC cells. Apoptosis of HCC cells after treatment with tBHP (25 μmol/L), SAHA (1 μmol/L), MS275 (1 μmol/L), or combinatorial treatment with tBHP and HDACi. (**a**) A single treatment with tBHP, SAHA, or MS275 showed a negligible effect on HepG2 cell apoptosis. Co-treatment with tBHP and HDACi dramatically increased apoptosis. (**b**) Hep3B cell death negligibly changed by a single treatment with tBHP, SAHA, or MS275. Both apoptotic and necrotic Hep3B cell populations drastically increased by combinatorial treatment with tBHP and HDACi. The mean of three independent FACS results from HepG2 (**c**) and Hep3B (**d**) was presented as stack columns. Different letters indicate significant differences at *p* < 0.05. ANOVA analyses of necrotic cells and late apoptotic cells are denoted using uppercase and lowercase letters, respectively. (**e**) Cleavage of caspase 12 was detected by Western blot.

It is well known that excess and prolonged ER stress induces cancer cell apoptosis [23]. We analyzed whether combinatorial treatment with tBHP and HDACi induces apoptosis, which is related to ER stress. We previously tested the effect of tBHP from 12.5 μmol/L to 400 μmol/L on HCC cell lines, where no further decrement in cell viability was observed from 100 μmol/L of tBHP onward [20]. Since 25 μmol/L of tBHP reduced the viability of HCC cells by approximately 20%, we determined that 25 μmol/L of tBHP induces mild oxidative stress in HCC and chose this dose for the present study. A single treatment with tBHP (25 μmol/L) slightly increased late apoptotic cell populations (12.6%) in HepG2 cells. The effect of a single HDACi (1 μmol/L) treatment on cell death was negligible, but combining tBHP with SAHA or MS275 drastically increased late apoptotic HepG2 cells by 27.2% and 22.6%, respectively (Figure 4a). A similar cell death trend was also observed in Hep3B cells; tBHP partially increased late apoptotic cells (8.5%). While the effects of HDACi administration were minuscule compared to the vehicle control, combining tBHP with SAHA or MS-275 induced a dramatic increase in apoptosis by 45.9% and 35.5%, respectively. Interestingly, necrotic cells also increased by co-treatment with tBHP and MS-275 (Figure 4b). FACS results acquired from three independent experiments using HepG2 cells (Figure 4c) and Hep3B cells (Figure 4d) were summarized as stack columns. We analyzed the cleavage of caspase 12, which is activated by ER stress. A single treatment with tBHP weakly induced caspase 12 cleavage, which was augmented by co-treatment with HDACi (Figure 4e).

**Figure 5:**
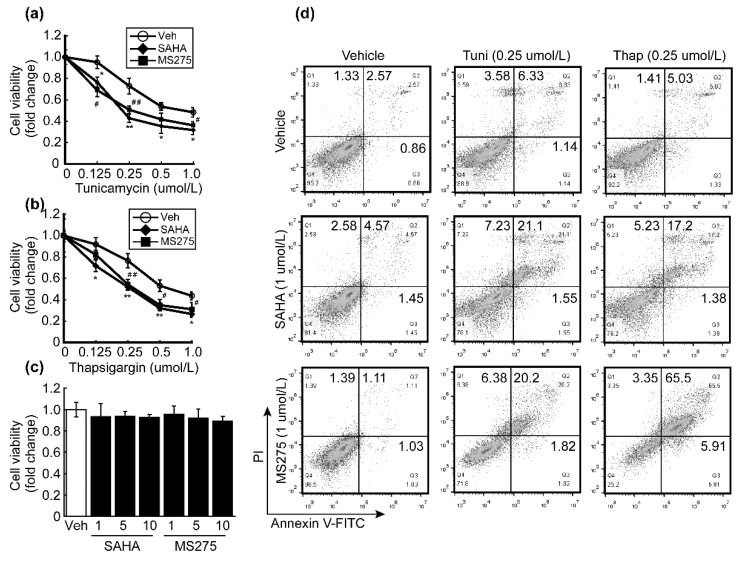
HDACi enhanced the effect of ER stressors. (**a**) Cell viability of HepG2 cells was analyzed after treatment with tunicamycin and/or pre-treatment with HDACi such as SAHA (1 μmol/L) and MS275 (1 μmol/L). Tunicamycin reduced the viability of HepG2 cells in a dose-dependent manner. Pre-treatment with HDACi enhanced the cytotoxicity of tunicamycin. (**b**) Thapsigargin decreased the viability of HepG2 cells, which was enhanced by pre-treatment with HDACi. Graphs show the mean ± SD of three independent experiments. (* *p* < 0.05, ** *p* < 0.01 vs. vehicle) (**c**) Cell viability was not affected by HDACi up to 10 μmol/L. (**d**) Cell death was analyzed by FACS. Cell death was negligibly changed by a single treatment with tunicamycin (0.25 μmol/L), thapsigargin (0.25 μmol/L), SAHA (1 μmol/L), or MS275 (1 μmol/L). Combining HDACi with either tunicamycin or thapsigargin drastically increased the apoptotic cell population of HepG2 cells.

We reconfirmed whether the apoptosis-inducing effect of HDACi is related to ER stress by using well-known ER stress inducers tunicamycin and thapsigargin. A single treatment with tunicamycin and thapsigargin decreased HepG2 cell viability in a dose-dependent manner. HDACi and ER stress inducers were synergistically associated with cytotoxicity in HCC. Combining 1 μmol/L of SAHA or MS-275 with tunicamycin (Figure 5a) and thapsigargin (Figure 5b) significantly lowered the viability of HepG2 cells, while HDACi treatment alone had a negligible impact on HepG2 cell viability (Figure 5c). To confirm the cell viability reduction and its relationship with apoptosis, FACS was conducted. Neither HDACi (1 μmol/L) nor ER stress inducers (0.25 μmol/L) alone induced significant changes in the apoptosis of HepG2 cells. However, combining HDACi with either tunicamycin or thapsigargin drastically increased the apoptotic cell population compared to respective controls (Figure 5d).

### 3.5. HDACi Facilitated Oxidative-Stress-Caused Reduction of Glutathione (GSH)

**Figure 6:**
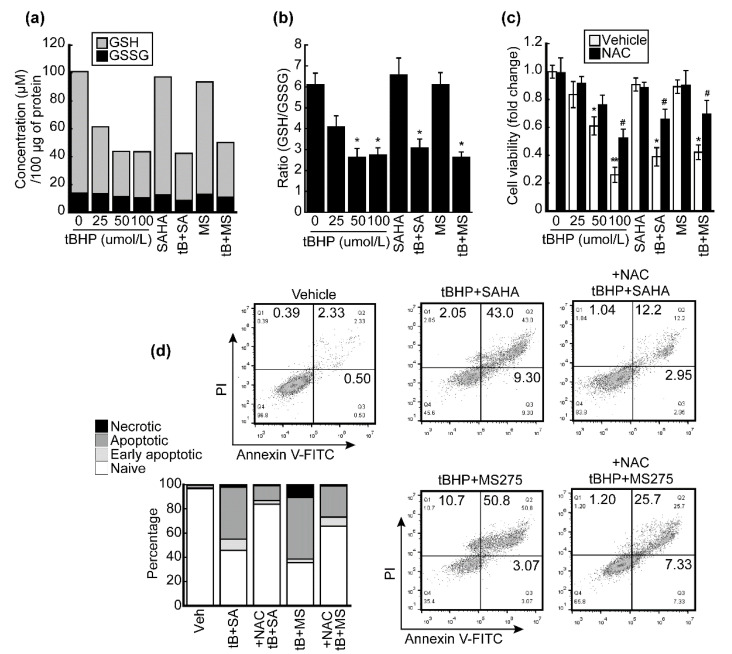
HDACi facilitated oxidative-stress-induced GSH reduction. (**a**) Intracellular GSH and GSSG levels were analyzed. The GSH level decreased by tBHP in a dose-dependent manner. HDACi-induced changes in GSH were negligible, while combining tBHP and HDACi resulted in substantial GSH reduction. Neither HDACi alone nor combinatorial treatment with tBHP and HDACi affected the GSSG level. (**b**) The ratio of intracellular GSH/GSSG was correlated with changes in GSH after tBHP, HDACi, or combined treatment. (**c**) Viability of HepG2 cells was analyzed using the CCK8 kit. The viability of HepG2 cells dose-dependently decreased by tBHP treatment, which was restored by pre-exposure to NAC (100 μmol/L). NAC partially reversed the HepG2 cell viability loss resulting from tBHP (25 μmol/L) and HDACi (1 μmol/L) co-treatment. (**d**) Cell death was analyzed by FACS. Combined treatment with tBHP and HDACi promoted the death of HepG2 cells compared to the vehicle. NAC pre-treatment decreased the cell death induced by combined treatment with tBHP and HDACi. Data were presented as the mean ± SD of three independent experiments (* *p* < 0.05, ** *p* < 0.01 vs. vehicle; # *p* < 0.05 tBHP + HDACi vs. NAC pre-treated).

Robust induction of the ER-stress-related gene Chac1 was observed following combinatorial treatment with tBHP and SAHA in HepG2 cells (Figure 2a). Since CHAC1 has a glutathione-specific gamma-glutamylcyclotransferase activity, which catalyzes the cleavage of GSH into 5-oxo-L-proline and cysteine-glycine dipeptide [24], we analyzed the changes in intracellular GSH levels after treatment with tBHP, HDACi, or both in HepG2 cells. The intracellular GSH level reduced by tBHP in a dose-dependent manner. HDACi (1 μmol/L)-induced changes to GSH levels in HepG2 cells were negligible, but combining HDACi with tBHP (25 μmol/L) resulted in synergistically decreased GSH levels. GSSG levels were unaffected irrespective of reagent treatment (Figure 6a); thus the ratio of intracellular GSH/GSSG was mainly determined by the GSH level (Figure 6b). HepG2 cell viability dose-dependently decreased upon tBHP treatment. While cell viabilities were unaffected by HDACi treatment alone, combined treatment using tBHP and HDACi halved the viability of HepG2 cells. The viability loss was recovered when cells were pre-treated with 100 μmol/L of N-acetyl-cysteine (NAC, Figure 6c). Consistent with this finding, HepG2 cells were subjected to apoptotic cell death upon exposure to tBHP and HDACi, but NAC pre-treatment suppressed this process (Figure 6d).

### 3.6. GPX8 Overexpression Restored Oxidative Stress and HDACi-Induced ER Stress and Apoptosis

**Figure 7:**
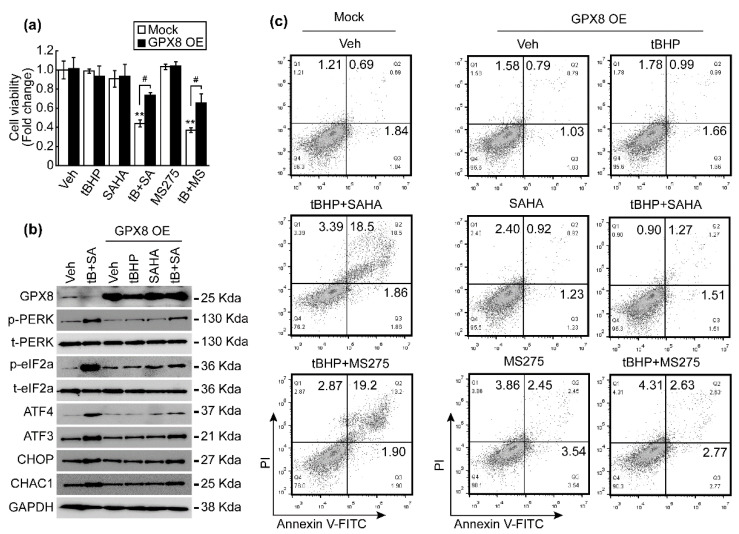
Overexpression of GPX8 reduced the ER stress and apoptosis induced by co-treatment with tBHP (25 μmol/L) and HDACi (1 μmol/L). (**a**) Cell viability was analyzed after treatment with tBHP, HDACi, or combined tBHP and HDACi in normal HepG2 and GPX8-overexpressed HepG2 cells. GPX8 overexpression reversed the cell viability loss resulting from tBHP and HDACi co-treatment. (**b**) Protein level of ER-stress-related genes was analyzed by Western blotting. Changes in expression of total PERK and eIF2a were negligible regardless of reagent treatment or GPX overexpression. Phospho-forms of PERK and eIF2a significantly increased by combined treatment with tBHP and SAHA, which was reversed by overexpression of GPX8. GPX8 overexpression reduced the co-treatment-induced increase in ATF4, ATF3, CHOP, and CHAC1 expression. (**c**) Cell death was analyzed by FACS. Overexpressing GPX8 inhibited the apoptosis induced through tBHP and HDACi co-treatment. Graphs showed the mean ± SD of three independent experiments (** *p* < 0.01 vs. vehicle, # *p* < 0.05 mock vs. GPX8 overexpression).

**Figure 8:**
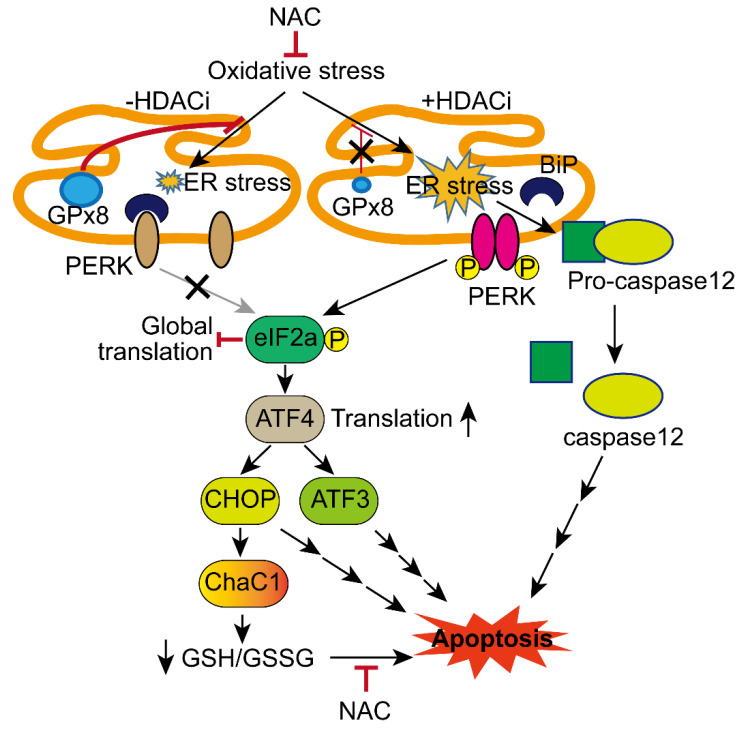
Summary of the present study. HDACi and oxidative stress reduced the expression of GPX8, which sensitized HCC cells to ER stress. Activation of the PERK/eIF2a/ATF4/CHOP/CHAC1 pathway and caspase12 by oxidative stress and HDACi exacerbated decrement of the GSH/GSSG ratio and cell death.

We investigated whether rescuing GPX8 reverses oxidative stress and HDACi-induced ER stress and apoptosis. GPX8 overexpression significantly restored the HepG2 cell viability loss incurred by tBHP (25 μmol/L) and HDACi (1 μmol/L) combinatorial treatment (Figure 7a). While total protein levels of PERK and eIF2a were unaffected regardless of drug treatment or GPX8 overexpression, changes to their phosphorylated forms were observed. Combinatorial treatment drastically increased the level of p-PERK and p-eIF2a, which were downregulated to basal levels by GPX8 overexpression. A similar trend was observed in ATF4, ATF3, CHOP, and CHAC1 upon combined treatment with tBHP and SAHA, whose expression was reduced by GPX8 overexpression (Figure 7b). Next, the effect of GPX8 overexpression on tBHP and HDACi-induced apoptosis in HepG2 cells was assessed. Co-treatment with tBHP and HDACi elevated the apoptotic cell populations of HepG2 cells, which was reversed by overexpressing GPX8 (Figure 7c). All of the results presented in this study are summarized in a schematic diagram in Figure 8.

## 4. Discussion

The present study demonstrated that HDACi treatment suppresses the expression of antioxidant enzymes, including GPX8, MGST, GSTO1, and NQO1, in HCC. Applying HDACi with mild oxidative stress to HCC drastically reduced GPX8 expression and activated a well-known ER stress response PERK/eIF2a/ATF4/CHOP/CHAC1 signaling pathway. CHAC1, as one of the final effectors of ER stress, contributed to cellular oxidative stress by decreasing GSH, a representative cellular antioxidant molecule that is also involved in the apoptosis of HCC cells.

ER is an intracellular organelle responsible for folding newly synthesized proteins mainly via disulfide bond formations, which are catalyzed by protein disulfide isomerases (PDI), ER oxidoreductin-1α (Ero1α), and peroxiredoxin4 (Prx4) [25]. GPX7 and GPX8 are ER-resident PDI oxidases lacking GSH and thioredoxin (Trx)-binding motifs, which use hydrogen peroxide to oxidize PDIs [26]. Since GPX7 has shown a closer homology with GPX4 than GPX8, it was named as a non-selenocysteine-containing phospholipid hydroperoxide (NPGPx). GPX7 contains a retention signal (REDL) in the C-terminal tail whose cleavage induces translocation from the ER lumen to the Golgi apparatus [27]. GPX8 is anchored in the ER membrane by a single transmembrane domain in the N-terminus, while the C-terminal portion of the protein faces the ER lumen [26]. While a single molecule of GPX7 showed higher binding affinity to H_2_O_2_ and PDIs than that by GPX8 [28], these results may have been determined using identical enzymatic concentrations of GPX7 and GPX8 in vitro. In natural settings, the endogenous GPX7 expression level is much lower than that of GPX8. Endogenous GPX8 was clearly detected in the membrane fraction of the ER, whereas GPX7 was hardly detected in naive cells [26]. In line with this notion, our RNA-seq data revealed that the normalized read counts (NRCs) of Gpx7 are approximately 300-fold less than those of Gpx8 in HCC cells (data not shown). Finally, the ER-stress-reducing role was revealed by overexpression of GPX8 in HCC cells. GPX8 overexpression reversed both cell viability reduction (Figure 7a) and increased apoptosis (Figure 7c) induced by combinatorial treatment with tBHP and HDACi. In addition, the activated PERK/eIF2a/ATF4 signaling pathway was also attenuated by overexpression of GPX8 (Figure 7b).

Previously, we demonstrated that HDACi treatment sensitizes HCC cells to oxidative stress, which leads to cell cycle arrest with a negligible effect on normal hepatocytes [20]. Based on this study, we surmised that HDACi may reduce the expression of antioxidant enzymes in HCC cells. Our MACE-Seq result showed that antioxidant enzyme expression of Gpx8, Mgst1 and Mgst2, Gsto1, Nqo1, and Prdx decreases, while the expression of others such as GSTA1, GSTM3, and GPX3 increases by HDACi or combined treatment with tBHP and HDACi (Figure 1a). The overall orchestrated change in expression observed in the present study was toward sensitization to oxidative stress in hepatocellular carcinoma cells. Adding mild oxidative stress induced a negligible change in GPX8 expression, whereas significantly downregulated GPX8 expression was observed at mRNA (Figure 1a–c) and protein levels (Figure 1e,f) when HCC cells were treated with HDACi alone or in conjunction with mild oxidative stress. Significant reductions in GPX8 mRNA and protein expression by HDACi treatment were not observed in normal hepatocytes (Figure 1d,g). Similar to our results, HDAC inhibitors reduced the expression of GPX8 protein in fibroblasts derived from a Niemann–Pick type C patient [29] and Gpx8 mRNA in SK-MEL-3 melanoma cells [30]. From these results, we speculated that downregulation of GPX8 may cause an increase in hydrogen peroxide and oxidative stress levels in the ER, which interrupts proper folding and maturation of secreted and membrane proteins by inducing aberrant disulfide bonds, as reported previously [31]. It is well established that HDACi hyperacetylates histones, which results in transcriptionally active euchromatin. However, it has been demonstrated that HDACi treatment does not always increase the gene expression in cells. Evidently, numerous studies have demonstrated downregulation of gene expression via HDACi treatment. The mechanisms underlying such multifaceted aspects of HDACi-regulated gene expression are diverse, and acetylation of non-histone proteins such as transcription factors are thought to play a critical role in HDACi-induced gene regulation [32]. Therefore, further investigations exploring the molecular mechanism underlying HDACi-repressed expression of GPX8 should be conducted in the near future.

To date, the anti-cancer effect of HDACi has been extensively investigated, and this contributed to elucidating the underlying mechanisms of tumor-cell-specific cell death. One of the mechanisms is HDACi-induced ER stress [33]. Accumulation of unfolded or misfolded proteins in the ER lumen activates unfolded protein response (UPR) signaling pathways in the cytosol. Three UPR pathways have been well established, which include translational regulation through the initial activation of PERK and two transcriptional regulations by inositol-requiring enzyme 1 (IRE1) and activating transcription factor 6 (ATF6) [34]. We focused on the PERK/eIF2a/ATF4 signaling pathway in the present study since its downstream genes such as PPP1R15A (GADD34), ATF3, CHOP, and CHAC1 are significantly induced by SAHA or combinatorial treatment with tBHP and SAHA (Figure 2a). While changes to mRNA and protein levels of PERK and eIF2a (Figure 2c,d,i) were negligible irrespective of chemical reagent treatment, their phosphorylation was markedly elevated by tBHP, HDACi, and the two combined (Figure 2i). Phosphorylation of PERK and eIF2a was negligibly changed by HDACi or combinatorial treatment in normal hepatocytes (Figure 2j). These results indicate that HDACi-induced PERK/eIF2a activation is more tumor specific than normal hepatocytes. When activated by phosphorylation, eIF2a generally suppresses the translation initiation of mRNA, but some notable exceptions to this list include GCN4 and ATF4 mRNAs [34]. ATF4 is a transcription factor belonging to the basic leucine-zipper (bZIP) family whose target genes have dual activities (i.e., cell survival and cell death). ATF4 induces the expression of genes involved in adaptation and stress relief, such as amino acid transporters, metabolic enzymes, redox balance, and ER chaperones. However, ATF4 also upregulates pro-apoptotic signaling proteins such as BH3-only B-cell lymphoma 2 (BCL-2)-interacting mediator of cell death (BIM), NOXA (Latin for damage), and p53 upregulated modulator of apoptosis (PUMA), while downregulating anti-apoptotic BCL-2. It is known that persisting stressful conditions within cells can lead to ATF4-mediated apoptosis [35]. As expected, a slight increase in Atf4 mRNA expression was observed by treating HepG2 cells with tBHP, HDACi, or both, which correlated with drastically enhanced protein expression of ATF4 and its downstream genes (Figure 2e–i). In addition, combinatorial treatment with tBHP and SAHA induced dramatic translocation of ATF4, ATF3, and CHOP from the cytosol into the nuclei (Figure 3a). To confirm the transcriptional activity of ATF4, enrichment of ATF4 on the promoters of target genes such as Atf3 [36], Chop [37], and Chac1 was investigated by ChIP assay. Furthermore, binding of CHOP on the Chac1 promoter [24] was also assessed. We investigated whether ATF4 and CHOP co-occupy the same locus in the Chac1 promoter, because cooperation of the ATF4–CHOP heterodimer has been correlated with TRB3, an ER-stress-induced gene involved in apoptosis [38]. Combinatorial treatment with tBHP and HDACi (SAHA and MS-275) significantly elevated ATF4-binding affinity on the promoters of Atf3, Chop, and Chac1 (Figure 3b–d). Similarly, co-treatment with tBHP and HDACi increased CHOP binding on the same locus previously occupied by ATF4 (Figure 3e).

Recently, it has been revealed that CHAC1/MGC4504, a downstream gene of the ATF4/ATF3/CHOP cascade, is a novel pro-apoptotic component of the UPR signaling pathway [39]. Our MACE-Seq results showed that co-treatment with tBHP and SAHA drastically induces Chac1 in HepG2 cells (Figure 2a). Thus, we investigated whether combined treatment with tBHP and HDACi induces apoptosis in HCC cells. Treatment with either tBHP or HDACi alone had a negligible impact on cell death of HepG2 and Hep3B cells, but combining these two reagents significantly elevated the late apoptotic cell population in both HCC cell lines (Figure 4). Moreover, it has been demonstrated that CHAC1 degrades GSH into 5-oxo-L-proline and cysteine-glycine dipeptide, which leads breast cancer cells to be highly susceptible to ER stress. Supplementing N-acetyl-cysteine (NAC) alleviated the amino-acid-deprivation-induced intracellular ROS levels, mitochondrial fragmentation, and cell death [40]. To confirm whether hese findings are applicable to the present study, we evaluated the changes in intracellular GSH and GSSG levels after treatment with tBHP, HDACi, or co-treatment. The intracellular GSH level, as well as the GSH/GSSG ratio, dose-dependently decreased by tBHP. Combined treatment also significantly decreased the GSH level and the GSH/GSSG ratio in HepG2 cells (Figure 6a,b). Cell viability reduction induced through tBHP and HDACi co-treatment was significantly reversed by NAC supplementation (Figure 6d), which is consistent with the markedly decreased apoptosis (Figure 6d).

We investigated whether HDACi enhances the effect of classical ER stressors such as tunicamycin and thapsigargin. Tunicamycin inhibits N-glycosylation on proteins to result in improper protein folding, while thapsigargin inhibits Ca^2+^-ATPase in the sarco-/endoplasmic reticulum, resulting in depletion of calcium in the lumen. These two compounds induce apoptosis through the induction of the UPR signaling pathway [41]. A single treatment with tunicamycin or thapsigargin decreased cell viability of HepG2 in a dose-dependent manner, whose cytotoxic effect was synergistically enhanced by the addition of HDACi such as SAHA or MS-275 (Figure 5a,b). Apoptosis appears to be the main cause of decreased cell viability, because combinatorial treatment with tunicamycin or thapsigargin with HDACi drastically elevated apoptotic cells (Figure 5d).

## 5. Conclusions

In conclusion, HDACi represses the expression of GPX8, rendering HCC cells to become more vulnerable to ER stress and apoptosis, even under mild oxidative stress conditions. Oxidative stress and HDACi-induced CHAC1, a downstream effector of the PERK/eIF2a/ATF4 signaling pathway, exacerbate intracellular oxidative stress by depleting GSH, which plays a role in HCC apoptosis induced by oxidative stress and HDACi, as summarized in Figure 8.

## Data Availability

Data is contained within the article and supplementary materials. Fastq from MACE was deposited in the National Center for Biotechnology Information (NCBI) Sequence Read Archive (SRA) repository (accession no. PRJNA678827; https://www.ncbi.nlm.nih.gov/Traces/study/?acc=PRJNA678827) (accessed on 6 April 2021).

## Supplementary Materials

The following are available online at www.mdpi.com/article/10.3390/antiox10101503/s1, Table S1: Primer sequences for qPCR, Table S2: Primer sequences for ChIP assay.
