# Supplementary material for: Glutathione Peroxidase 8 Suppression by Histone Deacetylase Inhibitors Enhances Endoplasmic Reticulum Stress and Cell Death by Oxidative Stress in Hepatocellular Carcinoma Cells
Source: Antioxidants (Basel). 2021 Sep 22;10(10):1503. doi: 10.3390/antiox10101503 (PMC8533003; doi:10.3390/antiox10101503)
Supplement: Supplementary file 1 [file antioxidants-10-01503-s001.zip › antioxidants-1372571-supplementary.pdf]

Supplementary data

Glutathione peroxidase 8 suppression by histone deacetylase inhibitors enhances endoplasmic reticulum stress and cell death by oxidative stress in hepatocellular carcinoma cells

Hae-Ahm Lee[1], Ki-Back Chu[2], Eun-Kyung Moon[3], Fu-Shi Quan[1, 3*]

[1]Medical Research Center for Bioreaction to Reactive Oxygen Species and Biomedical Science Institute, School of Medicine, Graduate school, Kyung Hee University, Seoul, Republic of Korea

[2]Department of Biomedical Science, Graduate School, Kyung Hee University, Seoul, Republic of Korea

[3]Department of Medical Zoology, School of Medicine, Kyung Hee University, Seoul, Republic of Korea

Running title: HDACi facilitate ER stress by oxidative stress

Conflicts of interest: The authors declare no potential conflicts of interest

*** Correspondence:**

Fu-Shi Quan

Department of Medical Zoology, Medical Research Center for Bioreaction to Reactive Oxygen Species and Biomedical Science Institute, Kyung Hee University, 26, Kyungheedae-ro, Seoul, 02447, Republic of Korea

Tel: +82-2-961-2302

Email: fsquan@khu.ac.kr

Table S1. Primer sequences for qPCR

Gene (Accession NO.)	Sequence (5' to 3')	Tm (°C)	Amplicon size (bp)
	Human		
Atf3 (CR450334)	F: GTGCCGAAACAAGAAGAAGG<br>R: TGGAGTCCTCCCATTCTGAG	60	184
Atf4 (CU012942)	F: AGATGACCTGGAAACCATGC<br>R: CCTGGTCGGGTTTTGTTAAA	60	155
Chac1 (NM001142776)	F: TTCTCAGTCCCTGCCTGTCT<br>R: CTGGTCAAGTTCCAGGATGG	60	165
Ddit3 (Chop) (AY880949)	F: GCGCATGAAGGAGAAAGAAC<br>R: CCAATTGTTCATGCTTGGTG	60	165
eif2a (BC011885)	F: TTCTAAACCGGTGGCTTCTG<br>R: ATTCTGCATTTGATGGCACA	60	167
Eikak3 (Perk) (BC126354)	F: ACATTCAAATGCAGCTGTGC<br>R: ATGTTGGATGGCTTGAGGTC	60	175
Gpx8 (BC029424)	F: GCCTTTGAAGTGAAGGATGC<br>R: AAAAGCCAACACGCTGAAGT	60	174
	Rat		
Gpx8 (NM_001106411)	F: GGAAGGATGGTGTCTCTGGA<br>R: AAACTGATTGCACGGGAAAG	60	165
Gapdh (NM_017008)	F: AAGGTCATCCCAGAGCTGAA<br>R: GTCCTCAGTGTAGCCCAGGA	60	183

Table S2. Primer sequences for ChIP assay

	Primer set for ChIP (5' to 3')	Tm (℃)	Amplicon size (bp)
Atf3 (Positive)	F: TGAGGGCTATAAAAGGGGTGATGC<br>R: GCGAGAGAAGAGAGCTGTGCAGTG	60	111
Atf3 (Negative)	F: CCGTTCCAAAGCGAAGAAGTAGGT<br>R: CTGTATTCGTGCCCAGAATGCTAGA	60	86
Chop (Positive)	F: AAGAGGCTCACGACCGACTA<br>R: ATGATGCAATGTTTGGCAAC	60	172
Chop (Negative)	F: CTGGCTCCTGTTGTTCTCTACC<br>R: CTCACCTCTTAAGACCCGAATG	60	170
Chac1 (Positive)	F: TGGAGCTGAACCAATCAGCG<br>R: GTGGGGCAAATCCCCTTTTG	60	96
Chac1 (Negative)	F: GCCAGGAGCAAGATCAGGTT<br>R: CTCCCACCCTGACCAAAGTC	60	76
